# Genome Sequence of the Filamentous Actinomycete *Kitasatospora viridifaciens*

**DOI:** 10.1128/genomeA.01560-16

**Published:** 2017-02-09

**Authors:** Karina Ramijan, Gilles P. van Wezel, Dennis Claessen

**Affiliations:** Sylvius Laboratories, Molecular Biotechnology, Leiden University, Leiden, The Netherlands

## Abstract

The vast majority of antibiotics are produced by filamentous soil bacteria called actinomycetes. We report here the genome sequence of the tetracycline producer “*Streptomyces viridifaciens*” DSM 40239. Given that this species has the hallmark signatures characteristic of the *Kitasatospora* genus, we previously proposed to rename this organism *Kitasatospora viridifaciens*.

## GENOME ANNOUNCEMENT

Filamentous actinomycetes are among the most potent antibiotic producers. They have a complex life cycle that starts with the germination of spores, which then grow out to form a vegetative mycelial network. When nutrients become scarce, a developmental program is initiated, leading to the formation of aerial hyphae that differentiate into chains of uninucleoid spores (1, 2). These spores are better equipped to withstand harsh environmental conditions and can reinitiate growth in more favorable environments. The genera *Streptomyces* and *Kitasatospora* belong to the family *Streptomycetaceae* (3), members of which have a similar mycelial lifestyle and, as such, are difficult to discriminate. Here, we report the genome sequence of the tetracycline producer *“Streptomyces viridifaciens”* DSM 40239.

*Streptomyces viridifaciens* DSM 40239 was obtained from the German Collection of Microorganisms and Cell Cultures (DSMZ) and grown in Tryptic Soy Broth medium containing 10% sucrose until mid-exponential phase (4). Next, chromosomal DNA was isolated as described previously (4) and sequenced by BaseClear (Leiden, The Netherlands) using a combined Illumina/PacBio sequencing approach. The quality of the Illumina FASTQ sequences was enhanced by trimming off low-quality bases and assembled into contigs using CLC Genomics Workbench (version 8.0). The optimal k-mer size was automatically determined using kMerGenie (5). Contigs were organized into scaffolds based on the alignment of the PacBio continuous long reads (CLR) using BLASR (6). From the alignment, the orientation, order, and distance between the contigs were estimated using the SSPACE-LongRead scaffolder version 1.0 (7). Gapped regions within the superscaffolds were (partially) closed in an automated manner using GapFiller version 1.10 (8). The resulting genome sequence contains 9,560,682 bp organized into four scaffolds, with 112 gaps. The overall G+C content is 72.20%. The major part of the chromosome is contained on scaffold 2, consisting of 7,834,366 bp and with a total of 7,143 coding sequences (CDSs). In addition, we detected that scaffold 1 is a putative megaplasmid, here termed KVP1, consisting of 1,710,701 bp and carrying 1,516 genes. Consistent with other linear plasmids described for *Streptomyces*, KVP1 contained an autonomous replication origin that is centrally located and which contains two genes encoding a putative replication protein (BOQ63_04065) and a plasmid DNA primase/helicase-like gene (BOQ63_04060) (9, 10). Furthermore, KVP1 contains a *traA* gene for conjugative transfer (BOQ63_00350), as well as *parA* (BOQ63_03875) and *parB* (BOQ63_03880) genes required for DNA segregation. Analysis of biosynthetic gene clusters (BGCs) for natural products using antiSMASH 3.0 located 12 BGCss on KVP1 and 34 clusters on the chromosome (11). One of the BGCs showed very high homology to the BGC for chlortetracycline.

Detailed analysis indicated that the sequenced *S. viridifaciens* strain had hallmark signatures that are characteristic of members of the genus *Kitasatospora*. The *S. viridifaciens* sporulation protein SsgB, which can be used as a reliable marker to discriminate morphologically complex actinomycetes, is more closely related to homologues identified in sequenced *Kitasatospora* strains (12). Furthermore, the *bldB*, *whiJ*, and *mbl* genes are absent, all of which are invariably present in members belonging to the *Streptomyces* genus (13). As such, we previously proposed to reclassify *S. viridifaciens* as a genuine *Kitasatospora* strain and rename it *Kitasatospora viridifaciens* (13).

### Accession number(s).

The genome sequence has been deposited at DDBJ/ENA/GenBank under the accession no. MPLE00000000. The version described in this paper is version MPLE01000000.
